# Interaction of background genetic risk, psychotropic medications, and primary angle closure glaucoma in the UK Biobank

**DOI:** 10.1371/journal.pone.0270530

**Published:** 2022-06-28

**Authors:** Sayuri Sekimitsu, Jiali Wang, Tobias Elze, Ayellet V. Segrè, Janey L. Wiggs, Nazlee Zebardast

**Affiliations:** 1 Tufts University School of Medicine, Boston, MA, United States of America; 2 Department of Ophthalmology, Massachusetts Eye and Ear, Harvard Medical School, Boston, MA, United States of America; 3 Ocular Genomics Institute, Harvard Medical School, Boston, MA, United States of America; 4 Schepens Eye Research Institute, Harvard Medical School, Boston, MA, United States of America; Oregon Health and Science University, UNITED STATES

## Abstract

**Background/Aims:**

Psychotropic medications have been reported as a risk factor for angle closure disease. However, the interaction between background genetic risk for primary angle closure glaucoma (PACG) and susceptibility to angle closure disease among psychotropic medication users has not been investigated. Here we demonstrate the utility of a genome-wide polygenic risk score (PRS) in identifying and risk-stratifying subjects with PACG and investigate the association between PACG genetic burden and exposure to psychotropic medications on prevalent angle closure.

**Methods:**

This analysis used the UK Biobank dataset, a prospective cohort study of 502,506 UK residents. We constructed a PACG PRS for participants using genome-wide association study summary statistics from a multiethnic meta-analysis using the Lassosum method.

**Results:**

Among the 441,054 participants, 959 (0.22%) were identified as PACG cases. Individuals with PACG had higher PRS compared to those without PACG (0.24±1.03 SD vs. 0.00±1.00 SD, p<0.001) and PACG prevalence increased with each decile of higher PRS. Among individuals using psychotropic medication, those with PACG had higher average PRS (0.31±1.00 SD vs. 0.00±1.00 SD, p<0.001) and were more likely to have a PRS in upper deciles of polygenic risk (p = 0.04). At each decile of PRS, psychotropic medication use was associated with increased risk of PACG. These effects were more pronounced and significant in higher deciles.

**Conclusion:**

We demonstrate the utility of a PRS for identifying individuals at higher risk of PACG. Additionally, we demonstrate an important relationship where the association between psychotropic medications use and PACG diagnosis varies across the polygenic risk spectrum.

## Introduction

Glaucoma is the leading cause of irreversible blindness worldwide and primary angle closure glaucoma (PACG) accounts for nearly half of glaucoma related blindness. PACG affects 17.14 million people older than 40 worldwide, and is estimated to increase to 26.26 million by 2050 [1]. Compared to individuals with primary open angle glaucoma (POAG), individuals with PACG have a three-fold excess risk of severe, bilateral visual impairment [2].

PACG is a complex and heterogenous disease. It is also highly heritable; siblings of individuals with angle closure have 13.6 times greater odds of angle closure compared to siblings of individuals with open angles [3]. Narrow angles, historically considered a risk factor for PACG, is estimated to have a heritability of almost 60% in certain populations [4]. Recent multi-ethic genome-wide association studies (GWAS) have identified multiple loci associated with PACG, including *PLEKHA7*, *COL11A1*, and *PCMTD1–ST18* [5, 6]. Genes located within associated loci are expressed within PACG-related structures (iris, ciliary body and the choroid), the blood-aqueous barrier structures (posterior iris epithelium, iris and ciliary body microvasculature), and type XI collagen (component of interstitial extracellular matrix), amongst others [7, 8]. For diseases with complex inheritance such as PACG, polygenic risk, the aggregate risk arising from multiple common genetic variants of individual small effect, can be calculated and associated with disease risk and outcome [9]. To date, genome-wide polygenic risk scores (PRSs) have been used to identify individuals at high genetic risk for many diseases including coronary artery disease, atrial fibrillation, and POAG [10]. While prior genetic risk scores have been created and evaluated, a genome-wide PRS has not yet been calculated or evaluated for PACG [11, 12].

In addition to genetic influences, environment triggers such as medications can predispose to angle closure glaucoma [13]. Adrenergic agonists, anticholinergics, and serotonergic medications have been implicated in triggering angle closure through pupillary block or anterior rotation of the lens/ciliary body complex [14]. Psychotropic medications have been reported to trigger angle closure and angle-closure glaucoma [13–17]. For example, serotonergic medications like selective serotonin reuptake inhibitors (SSRIs), serotonin and norepinephrine reuptake inhibitors (SNRIs), monoamine oxidase inhibitors (MAOis), tricyclic antidepressants (TCAs) stimulate 5-hydroxytryptamine receptors causing relaxation of the iris sphincter muscles, mydriasis, and increased production of aqueous humor [16]. Susceptibility to the effects of these medications may be partly genetically mediated and gene-environment interactions may explain some aspects of PACG pathophysiology.

The relationship between background PACG genetic risk and susceptibility to angle closure among psychotropic medication users has not been previously investigated. The purpose of our study was to understand the utility of a genome-wide PRS in identifying and risk-stratifying subjects with PACG and to investigate the association of PACG genetic burden and exposure to psychotropic medications on prevalent angle closure. Insights from these analyses could provide an opportunity to identify patients at high risk for PACG and could reinforce the importance of genetic risk profiling in clinical medicine.

## Materials and methods

### The UK Biobank

This analysis used the UK Biobank (UKBB) dataset, a prospective cohort study of 502,506 UK residents aged 40–69. Over 130,000 participants underwent eye examinations, including cornea-corrected intraocular pressure (IOPcc; Ocular Response Analyzer, Reichert, Depew, NY), autorefraction (RC-5000, Tomey, USA), corneal hysteresis (CH; Ocular Response Analyzer, Reichert, Depew, NY), and corneal resistance factor (CRF; Ocular Response Analyzer, Reichert, Depew, NY). The National Research Ethics Service Committee NorthWest–Haydock approved the study, and it was conducted in accordance with the Declaration of Helsinki. All participants provided written informed consent.

### Assessment of PACG and ocular factors

Individuals with PACG were identified by International Classification of Diseases, Ninth or Tenth Revision (ICD9/10) diagnosis code for PACG (ICD9: 365.2; ICD10: H40.2) from UKBB data field 41271/41270. Individuals with primary open angle glaucoma (POAG) were identified by ICD9/10 diagnosis codes for POAG, other glaucoma, or glaucoma unspecified (ICD9: 365.0, 365.1, 365.5, 365.6, 365.9; ICD10: H40.0, H40.1, H40.3-H40.6, H40.8, H40.9, H4.20, H4.28). Participants diagnosed with POAG, but not PACG and/or participants with a history of cataract surgery (UKBB data field 5324) were excluded from this analysis (n = 6,448).

IOPcc, CH, and CRF for the right and left eye were obtained from UKBB data fields 5254, 5262, 5256, 5264, 5257, and 5265 respectively. Information on IOPcc-lowering medication use was obtained from UKBB data field 20003; pre-treatment IOPcc was imputed by dividing IOPcc by 0.7 for those on IOP medication [18]. Spherical power and cylindrical power for the right and left eye were obtained from UKBB data fields 5084, 5085, 5087, and 5086 respectively. Spherical equivalent (SE) was calculated by adding half the cylindrical power to the spherical power. Outlier values (pre-treatment IOPcc values <5mmHg or >60mmHg and CH, CRF, and SE values greater than 3 standard deviations from the mean) were removed from analyses. Participant-level IOPcc, CH, CRF and SE values were calculated using measurements of the eye with higher IOPcc. If data was available for only one eye, data for that eye was used.

### Assessment of psychotropic medication use

Individuals using psychotropic medications were identified if they reported psychotropic medication use (UKBB data field 20003) (n = 38,646) or by ICD9/10 diagnosis codes for psychotropic disease (UKBB data field 41271/41270) (n = 24,247). We used ICD9/10 diagnosis codes for psychotropic disease as a proxy, assuming that these individuals currently or previously used psychotropic medications. Psychotropic medication classes include antipsychotics, anxiolytics, atypical antidepressants, atypical antipsychotics, bipolar medications, MAOis, SSRIs, SNRIs, TCAs, and topiramate (**S1 Table in S1 File**). Psychotropic diseases include schizophrenia, schizotypal and delusional disorders, mood disorders, and other anxiety disorders (**S2 and S3 Tables in S1 File**).

### PACG polygenic risk score calculation

The PRS for PACG for UKBB participants were computed using GWAS summary statistics from a multiethnic meta-analysis of PACG cases and controls [5]. Participants’ genetic data (hard calls, 533,176 variants) were utilized; variants with call rate < 97%, MAF < 0.01 and Hardy-Weinberg equilibrium test p < 1e-5 were removed as previously described by Kolli et al. [19]. To predict the ancestral background of participants using ancestral labels from the 1000 Genomes Project Phase 3 reference panel, Principal Component Analysis (PCA) to linkage disequilibrium (LD)-pruned (r2<0.1 in 200kb windows) genetic markers with minor allele frequency (MAF)>1% and the k-nearest neighbors algorithm were used [20]. The PRS was computed using the Lassosum method and implemented in R package lassosum. Lassosum uses a non-Bayesian regression-based model that shrinks variants via variable selection and retains the best set of variants by adjusting the tuning parameters [21]. Calculated PRS were normalized to a mean of zero and standard deviation (SD) of one for all further analyses.

### Statistical analysis

Statistical analyses were performed using RStudio v1.4.1106. Means and standard deviations were calculated for demographic and ocular characteristics. Means/frequencies were compared across groups using two-tailed Student’s t-tests and Chi-square tests/exact Fisher tests for continuous and categorical variables, respectively. We used logistic regression models adjusted for age, sex, and inferred ancestry to evaluate associations between PRS and psychotropic medication use with PACG diagnosis. Linear regression models were used to estimate associations between PRS decile and IOPcc, CH, CRF, and SE. We used the R package pROC to calculate the area under the curve (AUC) receiver operating curve [22]. P values less than 0.05 were considered statistically significant. Bonferroni adjustment was used to account for multiple hypothesis testing where appropriate.

## Results

### Cohort characteristics

Among the 441,054 UKBB participants included in this analysis, 959 (0.22%) were identified as PACG cases. 89,543 participants (90 cases, 89,543 controls) had ocular data, including IOPcc, CH, CRF, and SE.

The demographic and ocular characteristics among individuals with and without PACG are displayed in **Table 1**. Compared to controls, subjects with PACG were, on average, older (61.2±6.29 vs. 56.5±8.03 years, p<0.001) and more likely to be female (65.1% vs. 54.0%, p<0.001. On average, subjects with PACG also had higher medication adjusted IOPcc (22.0±8.01 mmHg vs. 16.8±3.19 mmHg, p<0.001), higher CRF (11.5±2.29 mmHg vs. 10.5±1.93 mmHg, p<0.001), and hyperopic refractive error (1.87±2.39 D vs. -0.21±2.39 D, p<0.001).

**Table 1:** Demographic/ocular characteristics and medication use of individuals with and without PACG.

	No PACG *n = 440*,*095*	PACG *n = 959*	p value
Age (SD)	56.5 (8.03)	61.2 (6.29)	<0.001
Ancestry:			0.874
Asian (%)	10,440 (2.37%)	24 (2.50%)	
European (%)	429,655 (97.6%)	935 (97.5%)	
Male sex (%)	202,515 (46.0%)	335 (34.9%)	<0.001
Polygenic risk score (SD)	0.00 (1.00)	0.24 (1.03)	<0.001
*Psychotropic medication use*			
Using psychotropic medications (%)	51,527 (11.7%)	173 (18.0%)	<0.001
Using antipsychotic medications (%)	1,124 (2.18%)	5 (2.94%)	0.102
Using anxiolytic medications (%)	3,939 (7.64%)	18 (10.40%)	0.002*
Using atypical antidepressant medications (%)	1,825 (3.54%)	9 (5.20%)	0.021
Using atypical antipsychotic medications (%)	1,184 (2.29%)	9 (5.20%)	0.001*
Using bipolar medications (%)	3,351 (6.50%)	9 (5.20%)	0.657
Using MAOi medications (%)	158 (0.30%)	1 (0.58%)	0.293
Using SNRI medications (%)	2,442 (4.74%)	4 (2.31%)	0.722
Using SSRI medications (%)	18,378 (35.7%)	48 (27.7%)	0.230
Using TCA medications (%)	11,215 (21.8%)	35 (20.2%)	0.040
Using topiramate (%)	230 (0.45%)	3 (1.73%)	0.015
IOPcc, medication adjusted, mmHg (SD)	16.8 (3.19)	22.0 (8.01)	<0.001
CH, mmHg (SD)	10.3 (1.78)	10.2 (2.13)	0.939
CRF, mmHg (SD)	10.5 (1.93)	11.5 (2.29)	<0.001
SE, diopters (SD)	-0.21 (2.39)	1.87 (2.39)	<0.001

Abbreviations: PACG = primary angle closure glaucoma; IOPcc = corneal-corrected intraocular pressure; CH = corneal hysteresis; CRF = corneal resistance factor; SD = standard deviation; SE = spherical equivalent.

Note: Bonferroni correction applied to psychotropic medication subclass analysis

* denotes p-values < 0.05 after correction. 90 PACG cases and 89,543 controls had available data for ocular characteristics. Medication subclass proportions are computed based on the total number of participants with psychotropic medication use for no PACG and PACG individuals.

### PACG PRS performance

**Fig 1:**
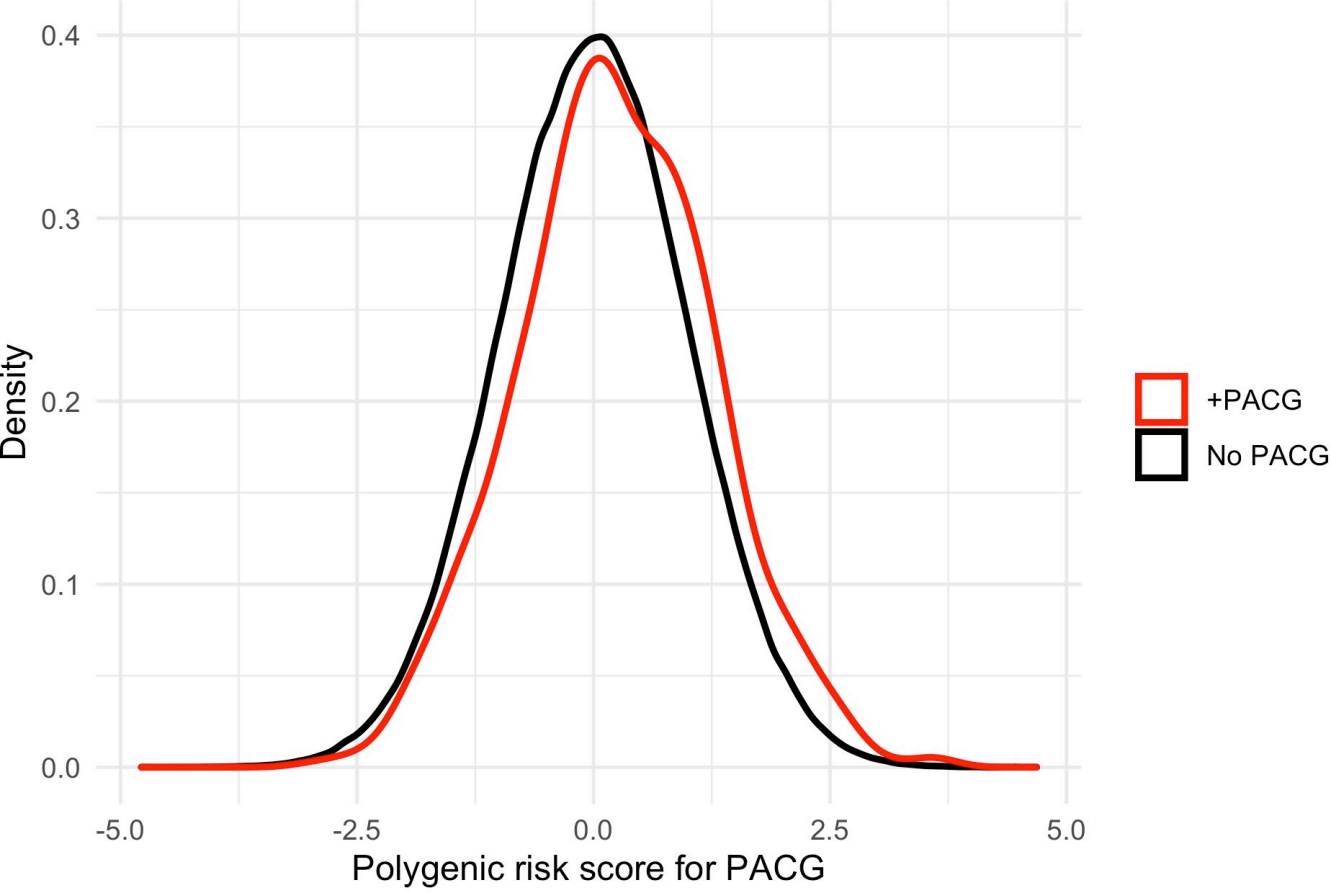
Distribution of PACG PRS amongst entire population.

**Fig 2:**
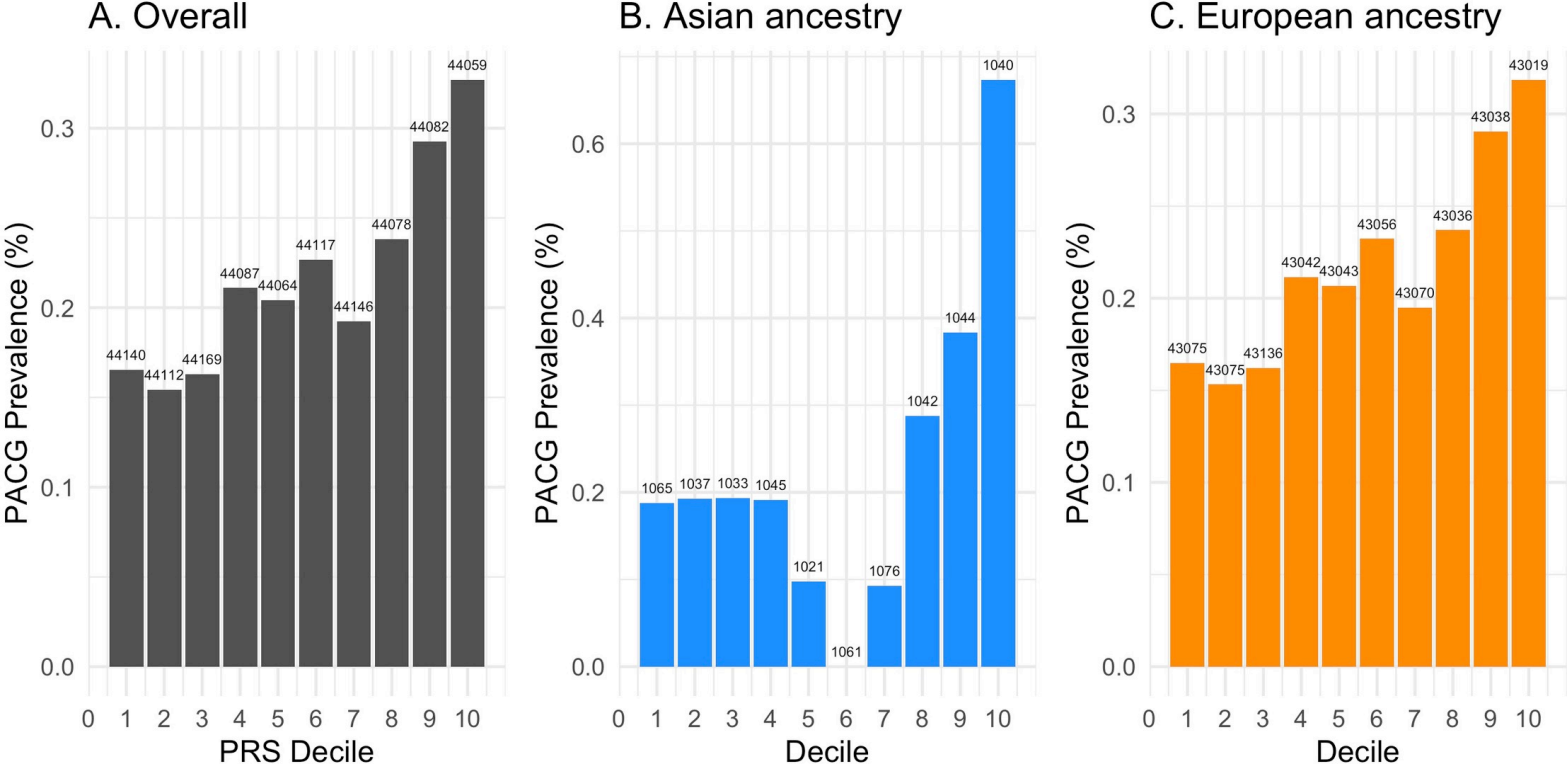
PACG prevalence by PRS decile. Note: number above each bar indicates the number of individuals in each decile.

A PACG PRS was computed for the 959 PACG cases and 440,095 controls. Individuals with PACG had significantly higher PRS for PACG compared to those without PACG (0.24±1.03 SD vs. 0.00±1.00 SD, p<0.001) (**Fig 1**). AUC receiver operative curve for PACG case detection in the entire population reached 0.68 for age and sex alone, 0.69 with the addition of PRS, and 0.819 with the addition of PRS and SE (**S1 Fig in S1 File**). When stratified by individuals of European and Asian ancestry, AUC reached 0.68 and 0.80 for age and sex alone, 0.69 and 0.82 with addition of PRS, and 0.81 and 0.99 with the addition of PRS and SE, respectively (**S2 Fig in S1 File**). The prevalence of PACG in decile 10 (those at highest genetic risk) was nearly double the prevalence of PACG in decile 1 (0.33 vs. 0.17%, p<0.001) (**Fig 2A**). When stratified by ancestry, a similar effect was observed in both Asian and European ancestry subsets of the population (**Fig 2B and 2C**). In a logistic regression model adjusting for age, sex, SE, ancestry, a one-point increase in PRS was associated with 1.30 times higher odds of PACG (95% CI, 1.05–1.59; p = 0.01) (**Table 2**).

**Table 2:** Regression analysis of predictors of PACG.

	Interval	OR (95% CI)	P-value
**Age**	1 year increase	1.10 (1.06–1.14)	<0.001
**Male sex**	vs. female	0.66 (0.42–1.02)	0.063
**Asian Ancestry**	vs. European	1.56 (0.47–3.76)	0.391
**Spherical equivalent (D)**	1 D increase	1.40 (1.28–1.54)	<0.001
**Polygenic risk score**	1 SD increase	1.30 (1.05–1.59)	0.014
**Psychotropic medication use**	vs. not using	1.95 (1.14–3.19)	0.010

Note: logistic regression includes all individuals with spherical equivalent measurements (n = 89,543 with 90 cases and 89,453 controls). Model includes age, sex, ancestry, polygenic risk score, and psychotropic medication use as covariates.

**Fig 3:**
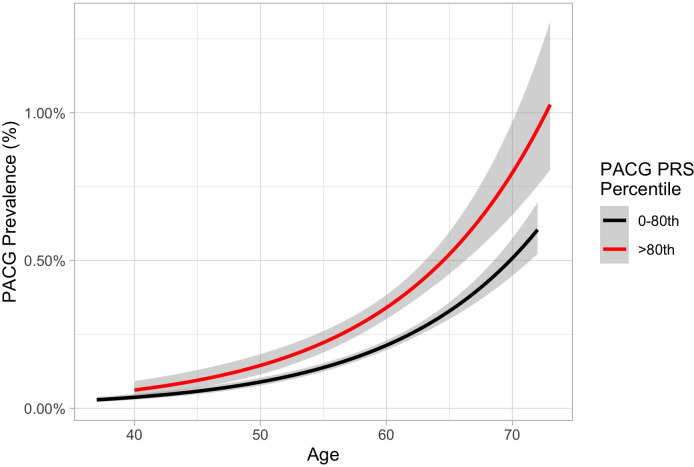
PACG prevalence by age, sex, and PRS percentile. Note: gray bars indicate 95% confidence interval.

As PACG prevalence is known to increase with advancing age, we performed stratified analyses by age and PRS (**Fig 3**). Individuals in the upper two deciles of polygenic risk for PACG had a higher prevalence of PACG compared to individuals in lower deciles of genetic risk at all ages. This effect was more pronounced in older individuals.

### Association of PACG PRS with ocular factors

Next, we looked at the association of PACG PRS with ocular factors. Changes in medication adjusted IOPcc and refractive error were seen by PACG PRS decile. For the entire cohort, in unadjusted models, each increase in PRS decile was associated with a 0.013±0.003 mmHg (p = 0.004) increase in IOPcc and a 0.015±0.003 D (p<0.001) increase in SE. In individuals without PACG, each increase in PRS decile was associated with a 0.013±0.003 mmHg (p<0.001) increase in IOPcc and a 0.015±0.003 D (p<0.001) increase in SE. Similarly, in individuals not using psychotropic medications, each increase in PRS decile was associated with a 0.013±0.004 mmHg (p<0.001) increase in IOP and a 0.014±0.003 D (p<0.001) increase in SE. Among individuals with PACG, no significant changes in IOPcc (p = 0.99) or SE (p = 0.34) were observed with increasing PRS. Among individuals using psychotropic medications, no significant change in IOPcc was observed with increasing PRS (p = 0.305), but each increase in PRS decile was associated with a 0.027±0.01 D (p<0.001) increase in SE. No significant changes in CH and CRF by PRS decile were observed.

### Association of PACG PRS, PACG PRS, and psychotropic medication use

Finally, we looked at the association between higher PACG PRS, PACG diagnosis, and psychotropic medication use. Compared to controls, individuals with PACG were more likely to be using psychotropic medications (18.0% vs. 11.7%, p<0.001) (**Table 1**). Subjects with PACG were more likely to be on anxiolytic (1.88% vs 0.90%, p = 0.002), atypical antidepressant (0.94% vs 0.41%, p = 0.021), atypical antipsychotic (0.94% vs 0.27%, p = 0.001), TCA (3.65% vs 2.55%, p = 0.04), and topiramate (0.31% vs 0.05%, p = 0.02) medications than controls. Differences in proportion of anxiolytic and atypical antipsychotic medication use survived Bonferroni adjustment. There were no significant differences in ancestry between the two groups, nor in use of other subclasses of psychotropic medications.

We found a significant association between PACG and psychotropic medication use among UKBB participants. In an unadjusted logistic regression model, psychotropic medication use was associated with 1.66 times higher odds of PACG (95% CI, 1.40–1.95; p<0.001). This association remained significant after adjusting for age, sex, ancestry, SE, and PRS (OR 1.95; 95% CI, 1.14–3.19; p = 0.01) (**Table 2**).

**Fig 4:**
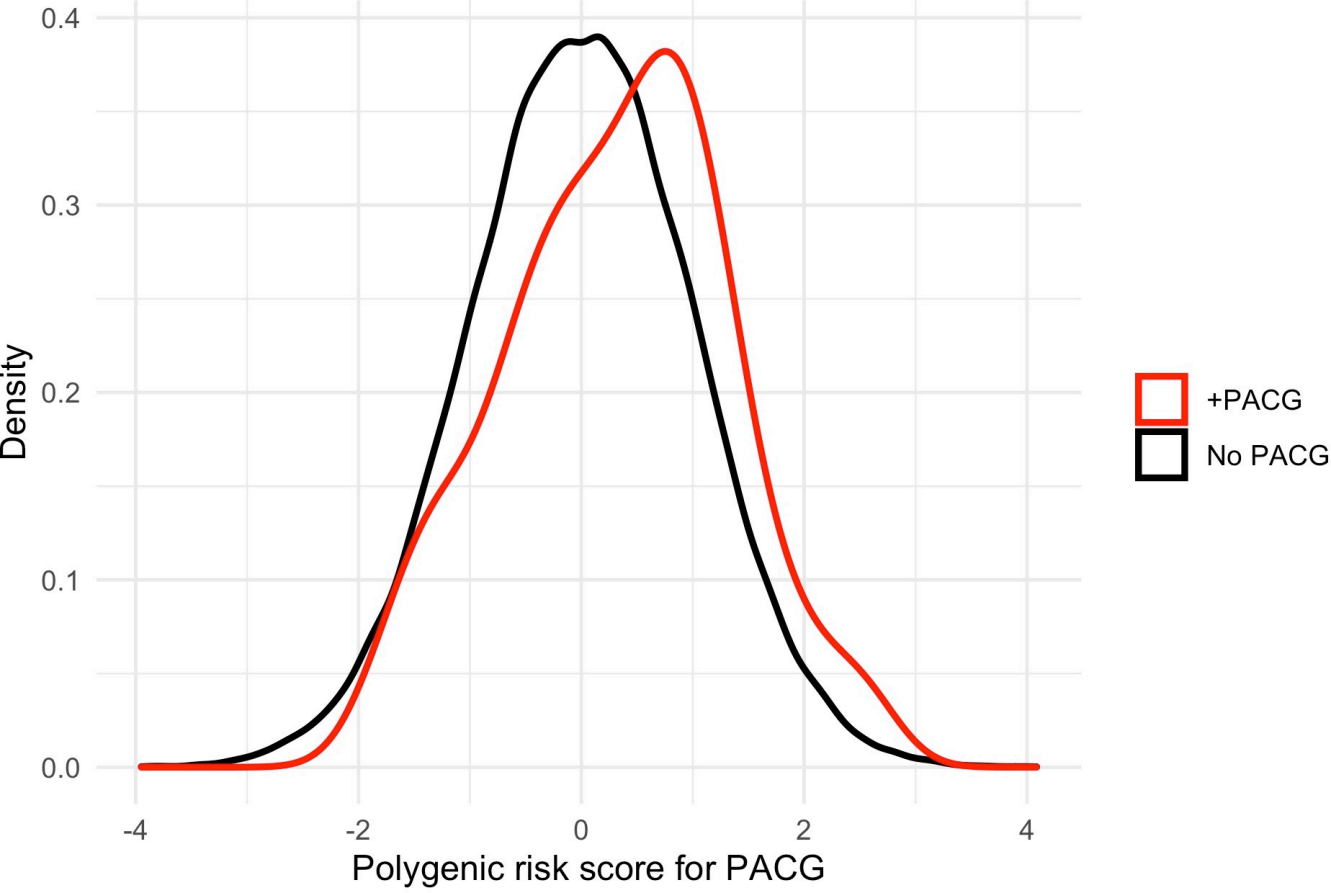
Distribution of PACG PRS amongst those who use psychotropic medications.

**Fig 5:**
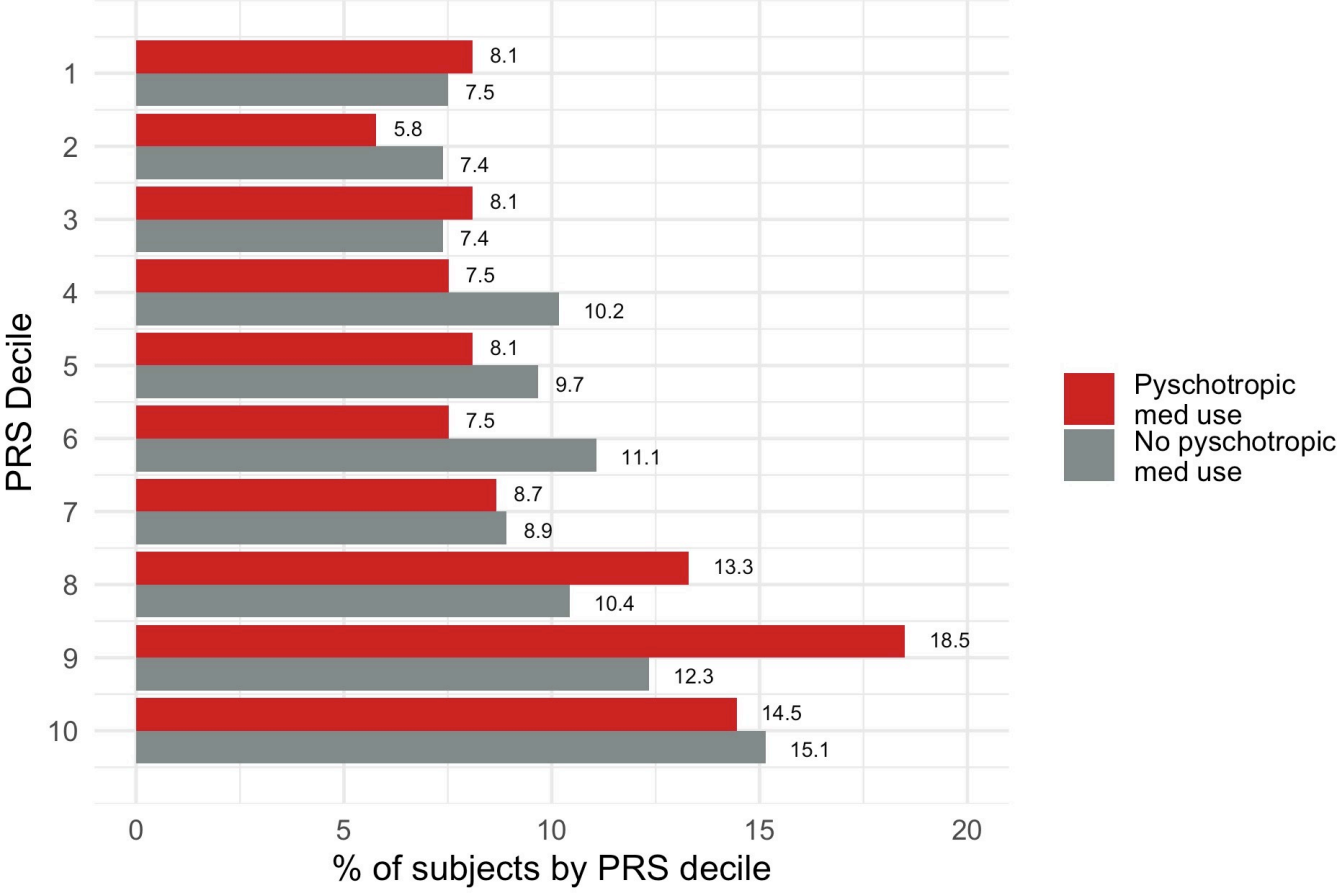
Proportion of PACG subjects who use or do not use psychotropic medications in each PRS decile.

To understand the interaction between PRS and psychotropic medication use on PACG prevalence, we stratified individuals according to medication usage. Among individuals using psychotropic medications, those with PACG had higher PRS compared to those without PACG (0.31±1.00 SD vs. 0.00±1.00 SD, p<0.001) (**Fig 4**). While PRS was slightly higher among individuals with PACG using psychotropic medications compared to individuals with PACG not using medications (0.31±1.00 SD vs 0.22±1.03 SD, p = 0.30), this difference was not significant. Individuals with PACG using psychotropic medications were more likely to have a PRS in decile 9 of PACG risk (18.5% vs. 12.3%, p = 0.04) (**Fig 5**).

**Fig 6:**
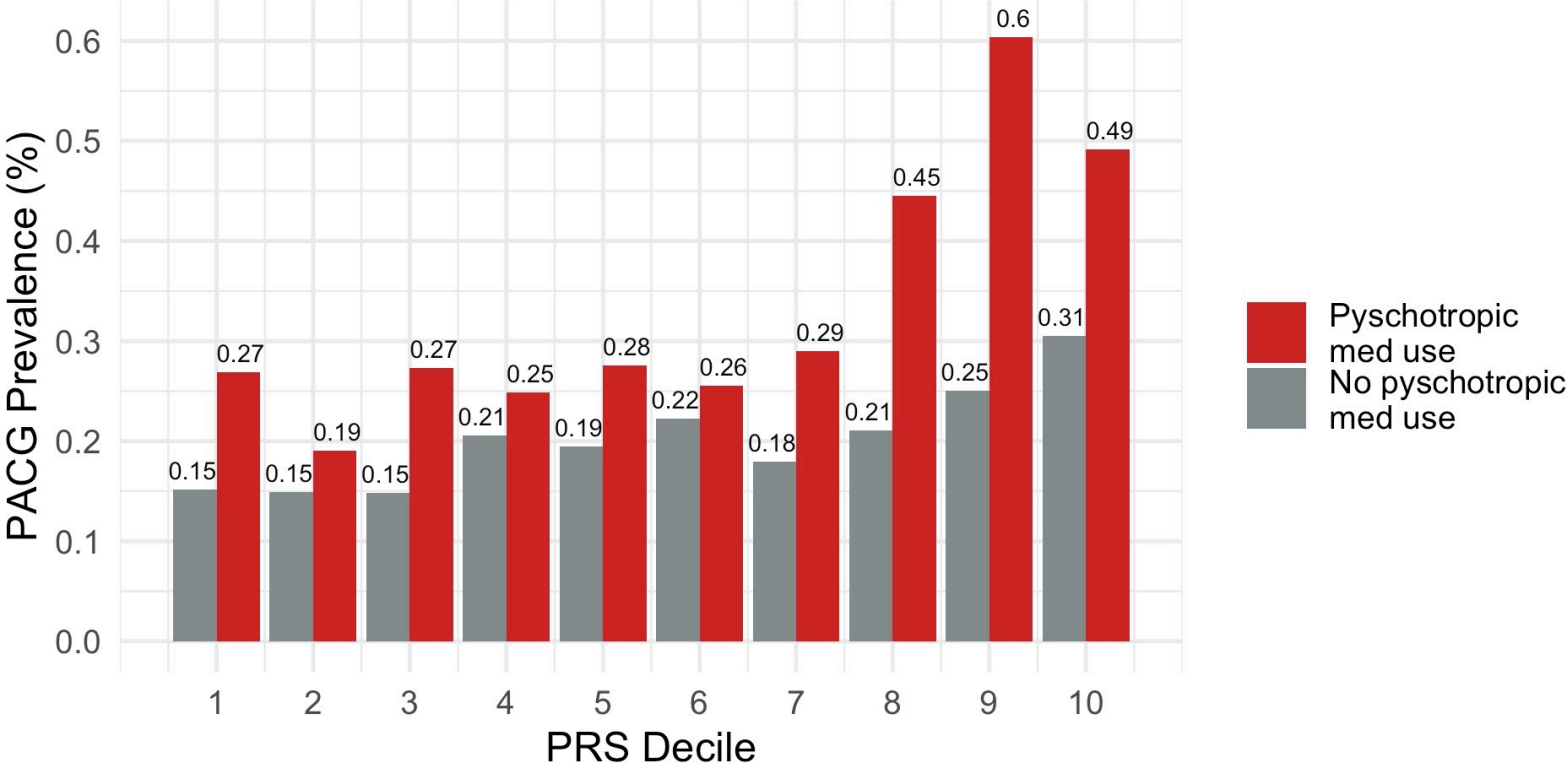
PACG prevalence per PRS decile and psychotropic medication use.

At each decile of polygenic risk, the use of psychotropic medications was correlated with increased PACG risk (**Fig 6**). These effects are more pronounced and significant in higher deciles. In decile 8, PACG prevalence was 2.1-fold higher for those on psychotropic medications versus those who were not (0.45% vs. 0.21%, p = 0.002). In decile 9, PACG prevalence was 2.4-fold higher for those on psychotropic medications versus those who were not (0.60% vs. 0.25%, p<0.001). And in decile 10, PACG prevalence was 0.49% for those on psychotropic medications and 0.31% for those who were not (p = 0.04).

## Discussion

Here we used data from a large-scale population-based study to demonstrate the utility of a genome-wide PACG PRS and to examine the influence of background genetic risk on prevalent PACG among those exposed to psychotropic medications, a known environmental risk. We found that in individuals of European and Asian ancestry, as polygenic risk increases, PACG prevalence increases. Interestingly, PACG PRS is associated with IOPcc and SE; as PRS increases, IOPcc increases and SE trends more hyperopic. Our data also show that psychotropic medication use is correlated with increased risk of PACG. Importantly, we demonstrate an important interaction where the association between psychotropic medications and PACG varies across the polygenic risk spectrum.

For the PACG PRS, we demonstrated good discretionary ability of genome-wide polygenic risk for PACG with AUC of 0.82 with addition of age, sex, and SE. Individuals with PACG were more likely to have a higher PRS, and PACG prevalence increased with each higher PRS decile. Importantly, we found that individuals with greater than 80th percentile of PACG PRS had a higher PACG prevalence at a younger age, which may indicate earlier disease presentation in those with highest genetic risk. While direct comparison is limited, in a Singaporean population, Nongpiur *et al*. previously reported AUC of 0.63 [12] for a PACG genetic risk score using 8 PACG-associated single nucleotide polymorphisms, similar to our estimate for the UKBB Asian population. It is important to note that we defined PACG using ICD codes in a population-based study, while Nongipur *et al*. identified PACG cases by clinical examination. It is plausible that our PRS would perform better when tested using prospective/clinical data and in populations with higher baseline risk. Nonetheless, the findings from our novel PACG PRS calculations reinforce that PACG is a polygenic disease with high heritability.

We also found that our PRS is associated with higher IOPcc and more hyperopic SE, both markers for potentially more severe disease. This effect was seen independent of PACG ICD codes and psychotropic medication use. The observed IOPcc association may in fact have a genetic basis: Wang *et al*. previously reported two PACG loci (*PLEKHA7* and *FERMT2*) to be associated with higher IOP [23]. It is possible that these two loci, in addition to other SNPs, are driving the observed association with IOPcc. Similarly, SE has been shown to be highly heritable and myopia is thought to be protective against PACG due to deeper anterior chamber depths [24–26]. This aligns with our finding that individuals with lower SE, thus more myopic, have a lower risk of PACG. In a large meta-analysis, Hysi *et al*. found one PACG locus (*ST18*) associated with myopia which, in addition to other SNPs, may be driving the observed trend with SE [27]. While both changes in IOPcc and SE are modest and likely have limited clinical significance, further investigation may elucidate our understanding of PACG pathogenic mechanisms.

As previously reported, use of psychotropic medications was associated with an independent increased risk of PACG in our cohort [13–17]. Previous research has shown that psychotropic medications increase the risk of PACG through changes in ciliary body position and pupillary block [14, 15]. Our analysis shows that individuals with PACG are more likely to use certain classes of drugs, anxiolytics, atypical antidepressants, atypical antipsychotics, TCAs, and topiramate. While other classes of medications like SSRIs previously reported to trigger angle-closure glaucoma did not reach statistical significance, this is possibly due to inadequate sample size [16, 17].

Importantly, background genetic risk is related to the environmental association between PACG and psychotropic medication use. Individuals with PACG using psychotropic medications have higher average PRS and were more likely to be in higher deciles of polygenic risk. While psychotropic medications use was associated with increased risk of PACG at every PRS decile, these effects were most pronounced at higher deciles. Altogether, our results suggest that there are genetic determinants that underly PACG development, as well as a physiologic ocular response to psychotropic drug exposure. Clinically, this may indicate that individuals who are on psychotropic medications and have a high polygenic risk may require more careful monitoring than those with low polygenic risk for PACG.

Our study is subject to several limitations. The summary statistics used to calculate our PACG PRS is derived from a largely Asian population and may not be entirely applicable to the UKBB population. As a result, the performance of our PRS is likely underestimated rather than overestimated, suggesting that the associations found here would only be strengthened by a European-specific PRS. Furthermore, previous research has shown that individuals of Asian and European ancestry tend to have similar PRS performance, so it is possible that this had a minimal impact [28]. We were unable to explore the interaction between polygenic risk and subclasses of psychotropic medications due to relatively low prevalence of PACG in the largely European UKBB cohort. Larger studies are needed to explore specific drug-gene interactions and resulting effects on PACG. Additionally, the lack of longitudinal and detailed clinical data precludes analysis of disease stage and determination of causal relationships. Though we show good discriminatory ability of our PRS, our use of ICD codes and self-reported medication use suffers possible ascertainment bias and imprecision inherit to such measures; however, the size of our dataset likely partially mitigates this problem. Also, the UKBB data only provides up to the first decimal of ICD codes, so we were unable to differentiate between acute angle-closure glaucoma from primary open angle glaucoma. Finally, our control population may have included undiagnosed PACG cases, though we attempted to account for this partially by excluding any participants with high IOPcc.

Here we demonstrate that higher polygenic risk and use of psychotropic medications independently increase PACG risk in both European and Asian populations. Our findings suggest an important interaction where higher PACG PRS is associated with the effect of psychotropic medications on PACG. This suggests that PRS may play an important role in optimizing PACG risk stratification among individuals on psychotropic medications, allowing for more careful disease monitoring and treatment. While further population-level, longitudinal research is needed to understand the relationship with genetic risk and environment triggers among angle closure patients, our results support the utility of polygenic risk scores in clinical medicine.

## Supporting information

S1 File (DOCX)
