# Supplementary material for: Interaction of background genetic risk, psychotropic medications, and primary angle closure glaucoma in the UK Biobank
Source: PLoS One. 2022 Jun 28;17(6):e0270530. doi: 10.1371/journal.pone.0270530 (PMC9239437; doi:10.1371/journal.pone.0270530)
Supplement: S1 File — (DOCX) [file pone.0270530.s001.docx]

**Supplementary Tables/Figures**

- **Supplemental Table 1.** Psychotropic medication list and codes
- **Supplemental Table 2.** Psychotropic disease list and ICD9 codes
- **Supplemental Table 3.** Psychotropic disease list and ICD10 codes
- **Supplemental Table 4.** Allele frequency distribution of top GWAS hits in UK Biobank cohort **Supplemental Fig 1.** Area under the receiver operating curve for PACG detection for polygenic risk score (PRS) with and without addition of age and sex.
- **Supplemental Fig 2a.** Area under the receiver operating curve for PACG detection for polygenic risk score (PRS) with and without addition of age and sex in individuals of Asian ancestry.
- **Supplemental Fig 2b.** Area under the receiver operating curve for PACG detection for polygenic risk score (PRS) with and without addition of age and sex in individuals of European ancestry.

**Supplemental Table 1.** Psychotropic medication list and codes

| **Drug code** | **Drug name** | **Category** |
| --- | --- | --- |
| 1140867150 | flupenthixol | Antipsychotic |
| 1140909800 | flupentixol | Antipsychotic |
| 1140867312 | melleril 10mg tablet | Antipsychotic |
| 1140868192 | buccastem 3mg tablet | Antipsychotic |
| 1140863410 | chloractil 25mg tablet | Antipsychotic |
| 1140867342 | clopixol 2mg tablet | Antipsychotic |
| 1140882320 | clozaril 25mg tablet | Antipsychotic |
| 1140866012 | combidol tablet | Antipsychotic |
| 1140910358 | cpz - chlorpromazine | Antipsychotic |
| 1140867210 | fentazin 2mg tablet | Antipsychotic |
| 1140867952 | fluanxol 500micrograms tablet | Antipsychotic |
| 1140882098 | fluphenazine | Antipsychotic |
| 1140867398 | fluphenazine decanoate | Antipsychotic |
| 1140867942 | fluphenazine hcl+nortriptyline 500micrograms/10mg tablet | Antipsychotic |
| 1140867940 | fluphenazine hydrochloride+nortriptyline 1.5mg/30mg tablet | Antipsychotic |
| 1140867184 | haldol 5mg tablet | Antipsychotic |
| 1140867168 | haloperidol | Antipsychotic |
| 1140863416 | largactil 10mg tablet | Antipsychotic |
| 1140867406 | loxapine | Antipsychotic |
| 1140867218 | pimozide | Antipsychotic |
| 1140867152 | depixol 3mg tablet | Antipsychotic |
| 1140867180 | dozic 1mg/ml oral liquid | Antipsychotic |
| 1140867084 | droperidol | Antipsychotic |
| 1140910356 | aminazine | Antipsychotic |
| 1140879658 | chlorpromazine | Antipsychotic |
| 1140909802 | levomepromazine | Antipsychotic |
| 1140867118 | methotrimeprazine | Antipsychotic |
| 1140867456 | modecate 12.5mg/0.5ml oily injection | Antipsychotic |
| 1140867156 | moditen 1mg tablet | Antipsychotic |
| 1140856004 | moditen enanthate 25mg/ml injection | Antipsychotic |
| 1140867136 | neulactil 2.5mg tablet | Antipsychotic |
| 1140867122 | nozinan 25mg tablet | Antipsychotic |
| 1140879754 | oxypertine | Antipsychotic |
| 1140867134 | pericyazine | Antipsychotic |
| 1140879674 | pipothiazine | Antipsychotic |
| 1140909804 | pipotiazine | Antipsychotic |
| 1140879746 | promazine | Antipsychotic |
| 1140868190 | proziere 5mg tablet | Antipsychotic |
| 1140867548 | redeptin 2mg/1ml injection | Antipsychotic |
| 1140867222 | rideril 25mg tablet | Antipsychotic |
| 1141177762 | risperdal 0.5mg tablet | Antipsychotic |
| 1140867474 | decazate 25mg/1ml oily injection | Antipsychotic |
| 1140927970 | serdolect 4mg tablet | Antipsychotic |
| 1140867092 | serenace 500micrograms capsule | Antipsychotic |
| 1141152860 | seroquel 25mg tablet | Antipsychotic |
| 1141184742 | solian 100mg/ml s/f oral solution | Antipsychotic |
| 1140867288 | sparine 50mg/5ml suspension | Antipsychotic |
| 1140867244 | stelazine 1mg tablet | Antipsychotic |
| 1140868172 | stemetil 5mg tablet | Antipsychotic |
| 1140856054 | taractan 15mg tablet | Antipsychotic |
| 1140879750 | thioridazine | Antipsychotic |
| 1140867208 | perphenazine | Antipsychotic |
| 1140868120 | trifluoperazine | Antipsychotic |
| 1140867332 | trifluperidol | Antipsychotic |
| 1140867334 | triperidol 500micrograms tablet | Antipsychotic |
| 1140855976 | veractil 25mg tablet | Antipsychotic |
| 1140872164 | zarontin 250mg capsule | Antipsychotic |
| 1140882100 | zuclopenthixol | Antipsychotic |
| 1140856296 | thiethylperazine | Antipsychotic |
| 1140856298 | torecan 6.33mg tablet | Antipsychotic |
| 1140863454 | buspar 5mg tablet | Anxiolytic |
| 1140879730 | buspirone | Anxiolytic |
| 1140863436 | equanil 200mg tablet | Anxiolytic |
| 1140863378 | meprobamate | Anxiolytic |
| 1140868170 | prochlorperazine | Anxiolytic |
| 1140855878 | tenavoid tablet | Anxiolytic |
| 1140863264 | trancopal 200mg tablet | Anxiolytic |
| 1141180638 | amfebutamone | Atypical antidepressant |
| 1141176854 | bupropion | Atypical antidepressant |
| 1140917466 | dutonin 100mg tablet | Atypical antidepressant |
| 1140917460 | nefazodone | Atypical antidepressant |
| 1140879634 | trazodone | Atypical antidepressant |
| 1141152732 | mirtazapine | Atypical antidepressant |
| 1140867806 | bolvidon 10mg tablet | Atypical antidepressant |
| 1140882244 | molipaxin 50mg capsule | Atypical antidepressant |
| 1141176858 | zyban 150mg m/r tablet | Atypical antidepressant |
| 1140867420 | clozapine | Atypical antipsychotic |
| 1141202024 | abilify 5mg tablet | Atypical antipsychotic |
| 1140867306 | dolmatil 200mg tablet | Atypical antipsychotic |
| 1140928916 | olanzapine | Atypical antipsychotic |
| 1141152848 | quetiapine | Atypical antipsychotic |
| 1140867444 | risperidone | Atypical antipsychotic |
| 1141153490 | amisulpride | Atypical antipsychotic |
| 1141195974 | aripiprazole | Atypical antipsychotic |
| 1140879704 | remoxipride | Atypical antipsychotic |
| 1140867432 | roxiam 150mg m/r capsule | Atypical antipsychotic |
| 1140927956 | sertindole | Atypical antipsychotic |
| 1140867304 | sulpiride | Atypical antipsychotic |
| 1140882376 | sulpitil 200mg tablet | Atypical antipsychotic |
| 1141185130 | sulpor 200mg/5ml oral solution | Atypical antipsychotic |
| 1141169714 | zotepine | Atypical antipsychotic |
| 1141167976 | zyprexa 2.5mg tablet | Atypical antipsychotic |
| 1140863392 | amylobarb sodium+quinalbarb sodium 50mg/50mg capsule | Anxiolytic |
| 1140882088 | amylobarbitone | Anxiolytic |
| 1140879696 | amylobarbitone sodium | Anxiolytic |
| 1140856008 | amytal 15mg tablet | Anxiolytic |
| 1140909724 | amobarbital | Anxiolytic |
| 1141180514 | amobarbital sodium | Anxiolytic |
| 1140909812 | methylphenobarbital | Anxiolytic |
| 1140872172 | methylphenobarbitone | Anxiolytic |
| 1140872180 | methylphenobarbitone 30mg tablet | Anxiolytic |
| 1140872134 | mysoline 250mg tablet | Anxiolytic |
| 1140910706 | phenobarbital | Anxiolytic |
| 1141181616 | phenobarbital product | Anxiolytic |
| 2038460068 | phenobarbitone | Anxiolytic |
| 1140872186 | phenobarbitone product | Anxiolytic |
| 1140872132 | primidone | Anxiolytic |
| 1140872174 | prominal 30mg tablet | Anxiolytic |
| 1140856018 | sodium amytal 60mg tablet | Anxiolytic |
| 1140863478 | soneryl 100mg tablet | Anxiolytic |
| 1140855856 | valium 10mg suppository | Anxiolytic |
| 1140863244 | valium 2mg tablet | Anxiolytic |
| 1140863250 | valium 2mg/5ml syrup | Anxiolytic |
| 1140855870 | almazine 1mg tablet | Anxiolytic |
| 1140863308 | alprazolam | Anxiolytic |
| 1140855930 | alupram 2mg tablet | Anxiolytic |
| 1140863318 | bromazepam | Anxiolytic |
| 1140863328 | chlordiazepoxide | Anxiolytic |
| 1140872150 | clonazepam | Anxiolytic |
| 1140855862 | anxon 15mg capsule | Anxiolytic |
| 1140863172 | dialar 2mg/5ml syrup | Anxiolytic |
| 1140863152 | diazepam | Anxiolytic |
| 1140855838 | evacalm 2mg tablet | Anxiolytic |
| 1140863110 | flurazepam | Anxiolytic |
| 1140863272 | frisium 10mg capsule | Anxiolytic |
| 1140855860 | ketazolam | Anxiolytic |
| 1140871578 | larapam 10mg capsule | Anxiolytic |
| 1140863320 | lexotan 1.5mg tablet | Anxiolytic |
| 1140863120 | loprazolam | Anxiolytic |
| 1140863302 | lorazepam | Anxiolytic |
| 1140863372 | medazepam | Anxiolytic |
| 1140863202 | temazepam | Anxiolytic |
| 1140855914 | triazolam | Anxiolytic |
| 1140855946 | centrax 10mg tablet | Anxiolytic |
| 1140910374 | clorazepate dipotassium | Anxiolytic |
| 1141157496 | diazepam product | Anxiolytic |
| 1140863350 | librium 5mg tablet | Anxiolytic |
| 1140863176 | lormetazepam | Anxiolytic |
| 1140855896 | nitrados 5mg tablet | Anxiolytic |
| 1140863182 | nitrazepam | Anxiolytic |
| 1140863374 | nobrium 5mg capsule | Anxiolytic |
| 1140855892 | noctamid 500micrograms tablet | Anxiolytic |
| 1140863210 | normison 10mg capsule | Anxiolytic |
| 1140863442 | oxazepam | Anxiolytic |
| 1140863274 | potassium clorazepate | Anxiolytic |
| 1140855944 | prazepam | Anxiolytic |
| 1140872152 | rivotril 500mcg tablet | Anxiolytic |
| 1140863106 | rohypnol 1mg tablet | Anxiolytic |
| 1140855900 | somnite 5mg tablet | Anxiolytic |
| 1140863238 | tensium 2mg tablet | Anxiolytic |
| 1140863276 | tranxene 7.5mg capsule | Anxiolytic |
| 1140863310 | xanax 250mcg tablet | Anxiolytic |
| 1141156650 | zileze 3.75 tablet | Anxiolytic |
| 1140872302 | lamictal 25mg tablet | Bipolar |
| 1140872290 | lamotrigine | Bipolar |
| 1141175204 | oxcarbazepine | Bipolar |
| 2038459704 | carbamazepine | Bipolar |
| 1140872064 | carbamazepine product | Bipolar |
| 1140872216 | convulex 150mg e/c capsule | Bipolar |
| 1140872198 | sodium valproate | Bipolar |
| 1140872072 | tegretol 100mg tablet | Bipolar |
| 1141167860 | teril cr 200mg m/r tablet | Bipolar |
| 1141185460 | teril retard 200mg m/r tablet | Bipolar |
| 1141175212 | trileptal 150 tablet | Bipolar |
| 1140872214 | valproic acid | Bipolar |
| 1141172838 | depakote 250mg e/c tablet | Bipolar |
| 1140867520 | li-liquid 5.4mmol/5ml oral solution | Bipolar |
| 1140867498 | liskonum 450mg m/r tablet | Bipolar |
| 1140867518 | litarex 564mg m/r tablet | Bipolar |
| 1140867490 | lithium product | Bipolar |
| 1140910976 | lithonate 400mg m/r tablet | Bipolar |
| 1140867494 | camcolit 250 tablet | Bipolar |
| 1140917270 | li-liquid 509mg/5ml oral solution | Bipolar |
| 1140867504 | priadel 200mg m/r tablet | Bipolar |
| 1140872348 | eldepryl 5mg tablet | MAOi |
| 1140867922 | manerix 150mg tablet | MAOi |
| 1140910504 | maoi - isocarboxazid | MAOi |
| 1140910704 | maoi - phenelzine | MAOi |
| 1140910820 | maoi - tranylcypromine | MAOi |
| 1140867858 | marplan 10mg tablet | MAOi |
| 1140856176 | marsilid 25mg tablet | MAOi |
| 1140867850 | phenelzine | MAOi |
| 1140879668 | selegiline | MAOi |
| 1140867914 | tranylcypromine | MAOi |
| 1140867944 | tranylcypromine+trifluoperazine 10mg/1mg tablet | MAOi |
| 1140856174 | iproniazid | MAOi |
| 1140867856 | isocarboxazid | MAOi |
| 1140867920 | moclobemide | MAOi |
| 1140867852 | nardil 15mg tablet | MAOi |
| 1140867932 | parstelin tablet | MAOi |
| 1140856344 | trancoprin tablet | MAOi |
| 1141169666 | zelapar 1.25mg tablet | MAOi |
| 1141151982 | edronax 4mg tablet | SNRI |
| 1140916288 | efexor 37.5mg tablet | SNRI |
| 1141201834 | cymbalta 30mg gastro-resistant capsule | SNRI |
| 1140916282 | venlafaxine | SNRI |
| 1141200564 | duloxetine | SNRI |
| 1141151978 | reboxetine | SNRI |
| 1140879688 | viloxazine | SNRI |
| 1140867770 | vivalan 50mg tablet | SNRI |
| 1140921600 | citalopram | SSRI |
| 1141190158 | cipralex 5mg tablet | SSRI |
| 1141151946 | cipramil 10mg tablet | SSRI |
| 1141180212 | escitalopram | SSRI |
| 1140867860 | faverin 50mg tablet | SSRI |
| 1140879540 | fluoxetine | SSRI |
| 1140879544 | fluvoxamine | SSRI |
| 1140867884 | lustral 50mg tablet | SSRI |
| 1140867888 | paroxetine | SSRI |
| 1140867878 | sertraline | SSRI |
| 1140855938 | oxanid 10mg tablet | SSRI |
| 1140851484 | paritane 20mg tablet | SSRI |
| 1140856212 | paynocil 600mg tablet | SSRI |
| 1140867876 | prozac 20mg capsule | SSRI |
| 1141185124 | ranflutin 20mg capsule | SSRI |
| 1140882236 | seroxat 20mg tablet | SSRI |
| 1140856074 | butriptyline | TCA |
| 1140856052 | chlorprothixene | TCA |
| 1140879624 | desipramine | TCA |
| 1140867600 | domical 10mg tablet | TCA |
| 1140867658 | elavil 10mg tablet | TCA |
| 1140867784 | ludiomil 10mg tablet | TCA |
| 1140879552 | maprotiline | TCA |
| 1140867824 | aventyl 10mg capsule | TCA |
| 1140867820 | allegron 10mg tablet | TCA |
| 1140879616 | amitriptyline | TCA |
| 1140867948 | amitriptyline hydrochloride+perphenazine 10mg/2mg tablet | TCA |
| 1140867938 | amitriptyline+chlordiazepoxide 12.5mg/5mg capsule | TCA |
| 1140867774 | amoxapine | TCA |
| 1140879620 | clomipramine | TCA |
| 1140867734 | concordin 5mg tablet | TCA |
| 1140879556 | mianserin | TCA |
| 1140909806 | dosulepin | TCA |
| 1140867930 | motival tablet | TCA |
| 1140879628 | dothiepin | TCA |
| 1140867818 | nortriptyline | TCA |
| 1140867618 | pertofran 25mg tablet | TCA |
| 1140856144 | praminil 10mg tablet | TCA |
| 1140867628 | prepadine 25mg capsule | TCA |
| 1140867640 | doxepin | TCA |
| 1141168396 | doxepin hydrochloride 5% cream | TCA |
| 1140867624 | prothiaden 25mg capsule | TCA |
| 1140879632 | protriptyline | TCA |
| 1140882310 | gamanil 70mg tablet | TCA |
| 1140879630 | imipramine | TCA |
| 1140867720 | iprindole | TCA |
| 1140882312 | sinequan 10mg capsule | TCA |
| 1140867758 | surmontil 10mg tablet | TCA |
| 1140867662 | lentizol 25mg m/r capsule | TCA |
| 1140856186 | limbitrol 10 capsule | TCA |
| 1140917388 | tranquax 10mg capsule | TCA |
| 1140867928 | limbitrol-5 capsule | TCA |
| 1140867726 | lofepramine | TCA |
| 1140867756 | trimipramine | TCA |
| 1140867668 | tryptizol 10mg tablet | TCA |
| 1141146062 | lomont 70mg/5ml s/f suspension | TCA |
| 1140923484 | topiramate | Topiramate |

**Supplemental Table 2.** Psychotropic disease list and ICD9 codes

| **Disease Category** | **Code** |
| --- | --- |
| Transient organic psychotic conditions | 293 |
| Schizophrenic psychoses | 295 |
| Affective psychoses | 296 |
| Other nonorganic psychoses | 298 |
| Neurotic disorders | 300 |
| Personality disorders | 301 |
| Depressive disorder, not elsewhere classified | 311 |

Note: See <https://biobank.ndph.ox.ac.uk/showcase/field.cgi?id=41271> for more detail.

**Supplemental Table 3.** Psychotropic disease list and ICD10 codes

| **Disease Category** | **Code** |
| --- | --- |
| Schizophrenia | F20 |
| Schizotypal disorder | F21 |
| Persistent delusional disorders | F22 |
| Acute and transient psychotic disorders | F23 |
| Induced delusional disorder | F24 |
| Schizoaffective disorders | F25 |
| Other nonorganic psychotic disorders | F28 |
| Unspecified nonorganic psychosis | F29 |
| Manic episode | F30 |
| Bipolar affective disorder | F31 |
| Depressive episode | F32 |
| Recurrent depressive disorder | F33 |
| Persistent mood [affective] disorder | F34 |
| Other mood [affective] disorder | F38 |
| Unspecified mood [affective] disorder | F39 |
| Phobic anxiety disorders | F40 |
| Other anxiety disorders | F41 |

Note: See <https://biobank.ndph.ox.ac.uk/showcase/field.cgi?id=41270> more detail.

**Supplemental Table 4.** Allele frequency distribution of top GWAS hits in UK Biobank cohort

| **Chromosome** | **ID** | **Major allele** | **Minor allele** | **Minor Allele Frequency: Asian** | **Minor Allele Frequency:**  **European** |
| --- | --- | --- | --- | --- | --- |
| 1 | rs3753841 | A | G | 0.399 | 0.388 |
| 7 | rs3816415 | G | A | 0.119 | 0.116 |
| 8 | rs1015213 | C | T | 0.086 | 0.090 |
| 9 | rs736893 | G | A | 0.285 | 0.346 |
| 9 | rs3739821 | A | G | 0.465 | 0.774 |
| 10 | rs1258267 | A | G | 0.056 | 0.034 |
| 11 | rs11024102 | T | C | 0.321 | 0.289 |
| 14 | rs7494379 | C | T | 0.356 | 0.301 |

**Supplemental Fig 1.** Area under the receiver operating curve for PACG detection for polygenic risk score (PRS) with and without addition of age and sex.

**
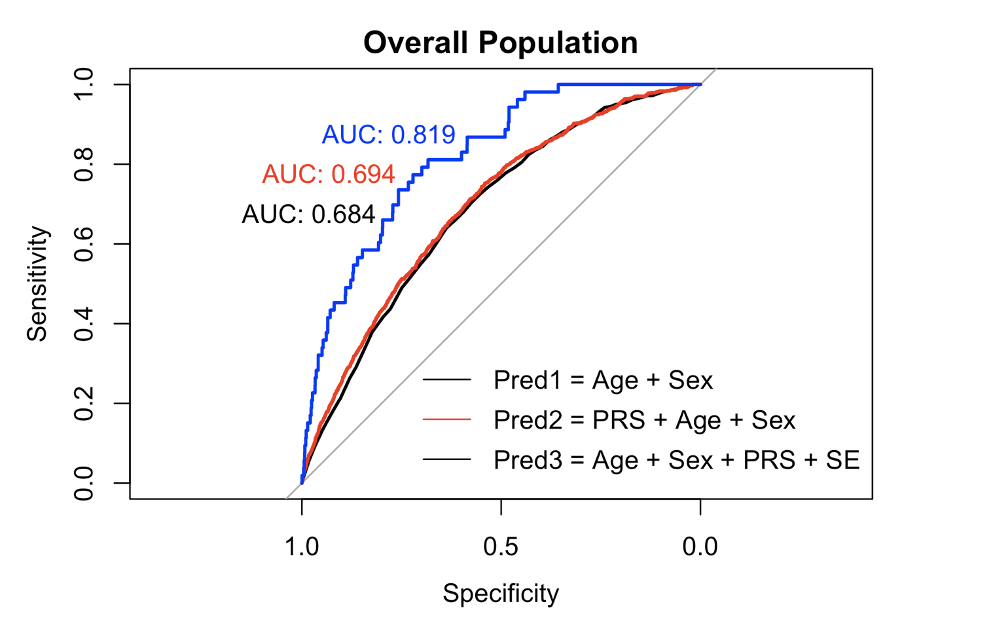
**

**Supplemental Fig 2a.** Area under the receiver operating curve for PACG detection for polygenic risk score (PRS) with and without addition of age and sex in individuals of Asian ancestry.

**
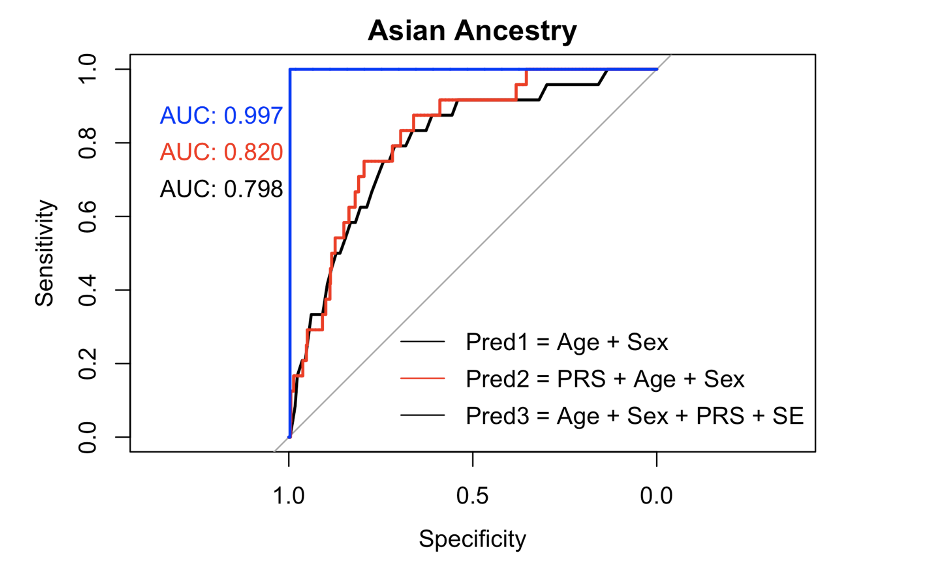
**

**Supplemental Fig 2b.** Area under the receiver operating curve for PACG detection for polygenic risk score (PRS) with and without addition of age and sex in individuals of European ancestry.

**
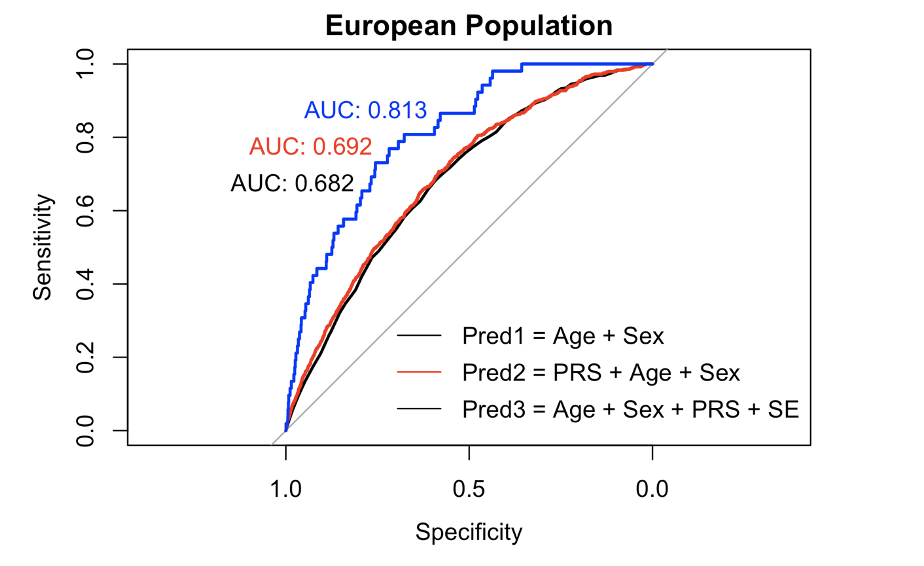
**
